# Differential impact of lytic viruses on prokaryotic morphopopulations in a tropical estuarine system (Cochin estuary, India)

**DOI:** 10.1371/journal.pone.0194020

**Published:** 2018-03-13

**Authors:** Vijayan Jasna, Angia Sriram Pradeep Ram, Ammini Parvathi, Telesphore Sime-Ngando

**Affiliations:** 1 CSIR-National Institute of Oceanography, Regional Centre (CSIR), Kochi, India; 2 Laboratoire Microorganismes: Génome et Environnement, UMR CNRS 6023, Université Clermont Auvergne, 1 Impasse Amélie Murat, Aubière, France; Universidade de Aveiro, PORTUGAL

## Abstract

Our understanding on the importance of viral lysis in the functioning of tropical estuarine ecosystem is limited. This study examines viral infection of prokaryotes and subsequent lysis of cells belonging to different morphotypes across a salinity gradient in monsoon driven estuarine ecosystem (Cochin estuary, India). High standing stock of viruses and prokaryotes accompanied by lytic infection rates in the euryhaline/mesohaline region of the estuary suggests salinity to have an influential role in driving interactions between prokaryotes and viruses. High prokaryotic mortality rates, up to 42% of prokaryote population in the pre-monsoon season is further substantiated by a high virus to prokaryote ratio (VPR), suggesting that maintenance of a high number of viruses is dependent on the most active fraction of bacterioplankton. Although myoviruses were the dominant viral morphotype (mean = 43%) throughout the study period, there was significant variation among prokaryotic morphotypes susceptible to viral infection. Among them, the viral infected short rod prokaryote morphotype with lower burst estimates (mean = 18 viruses prokaryote^-1^) was dominant (35%) in the dry seasons whereas a substantial increase in cocci forms (30%) infected by viruses with high burst size (mean = 31 viruses prokaryote^-1^) was evident during the monsoon season. Such preferential infections of prokaryotic morphopopulations with respect to seasons can have a strong and variable impact on the carbon and energy flow in this tropical ecosystem.

## Introduction

Viruses are ubiquitous biological entities that occur in a wide variety of aquatic ecosystems infecting both prokaryotes and eukaryotes [1, 2]. Through different life strategies (mainly lytic and lysogeny pathways), they play important roles in various ecological and biogeochemical, processes by regulating microbial diversity, carbon and nutrient fluxes, food web dynamics and mediating lateral gene transfers [3,4]. Viral-induced mortality of microorganisms can transiently remove up to 40% of daily prokaryotic production [5], which can substantially reduce the trophic transfer of carbon and nutrients in prokaryotic biomass to higher trophic levels [6,7].

During the last two decades, much attention has been given to viral mediated processes in marine coastal and oceanic waters and, to a lesser extent on transition zones such as estuaries especially from the tropics. The coastal oceans, especially tidally influenced estuaries, have drawn much attention as they are the most geochemically and biologically active areas of the biosphere which are characterized by steep salinity gradients, temperature and nutrient concentrations [8]. Estuarine habitats, perhaps the most exploited aquatic habitats, are of great interest for microbial ecologists as the mixing between marine tidal water and riverine discharge can trigger important physiological, genetic, and ecological shifts in microbial hosts, primarily bacteria [9, 10].

Among the tropical coastal ocean, Cochin estuary (southwest coast of India) is one of the largest and productive estuarine ecosystems which are strongly impacted by annually recurring monsoon cycles (wet season), where high precipitation keeps the estuary dominated by freshwater for about 6 months [11]. Multiple lines of evidences have suggested viruses as key players in microbial loop and viral shunt exerting their strong influence on the processes occurring in planktonic food web [12, 13]. Given the importance of viruses as significant players in regulating prokaryotic host dynamics in this net heterotrophic tropical estuarine system [12], we hypothesize that viruses could have a strong selective impact on the different morphotypes of prokaryotic populations with seasons (dry versus wet seasons). Study on the above aspect is crucial as variations in prokaryotic biomass (carbon pool) are strongly related to morphotype composition of prokaryotic populations [14]. Morphological adaptation by prokaryotes serves as an important biological function through cell surface-to-volume ratios which respond to environmental cues, and gain competitive advantage for coping with changing environment, especially in pelagic systems [15]. In order to enhance our understanding of aquatic microbial food webs, information on forces that determine the relative importance of viruses as a source of prokaryotic mortality among prokaryotic morphotypes is of considerable interest.

In the present study, variation in phage infectivity was examined in relation to environmental parameters, with an aim to improve our knowledge on viral dynamics in tropical estuarine systems. Variations in viral infection frequency and burst size were evaluated for different prokaryotic morphopopulations, in the context of total viral-mediated prokaryotic mortality and viral production. The findings of this study would provide crucial insight into the viral ecology and viral induced mortality in this tropical estuarine ecosystem which are strongly impacted by both tidal and monsoonal cycles.

## Materials and methodology

### Study site and sampling

A total of 13 stations in the Cochin Estuary (southwest coast of India) situated between 9° 30′ and 10° 20′ N and 76° 13′ to 76° 5′ E, (see Parvathi et al., for detailed site description and characteristics) [12] were selected for the study based on three different salinity regimes namely, Zone I (stations 1,2,6,7,8 and 9), Zone II (stations3,4 and 5) and Zone III (stations10,11,12, and 13) (**S1 Fig**). Surface water samples (0.5m depth) were collected using a 5L Niskin sampler (Hydro-Bios, Germany) in three distinct seasons, Pre monsoon (PRM; March-April), Monsoon (MON; July-August), and Post monsoon (PM; October-November) in 2014. All water samples, collected in triplicates were transferred to sterile containers and transported to laboratory in refrigerated boxes where they were processed immediately upon arrival.

### Physicochemical parameters

Temperature and salinity were measured using a conductivity temperature density profiler (CTD, SBE, Seabird 19) (accuracy ± 0.001°C for temperature and ± 0.001 S/m for conductivity). Salinity was also measured using an Autosal (Guild line) for correcting the salinity measured using CTD. Dissolved oxygen (DO) was estimated according to Winkler’s titration method. Dissolved inorganic nutrients such as ammonia (NH_4_-N), nitrite (NO_2_-N), nitrate (NO_3_-N), phosphate (PO_4_-P) and silicate (SiO_4_-Si) were analyzed spectrophotometrically according to standard procedures [16]. Chlorophyll *a* (Chl *a*) concentrations were determined fluorometrically from samples (500 ml) collected on GF/F filters (0.7 μm, Whatman, USA). The pigments were extracted in 90% acetone overnight in the dark at 4°C and concentrations were calculated as described in Parsons et al [17]. The supernatant was used to determine the functional Chl *a* pigments and acidified with 0.1 N HCl to estimate phaeophytin [17].

#### Viral abundance (VA), Prokaryotic abundance (PA) and Total viable counts (TVC)

For enumeration of viruses (VA) and prokaryotes (PA), water samples were fixed immediately with 0.02 μm filtered, buffered formalin (2% *v/v*). Subsamples of 1–2 ml were filtered (<15 KPa vacuum) through 0.02 μm pore-size Anodisc filters (Whatman, USA) and stained with 1:400 diluted SYBR green I (Invitrogen, CA, USA) as previously described [18]. The filters were air dried on absorbent paper and mounted between a slide and a glass cover slip with a special antifade solution consisting of 50%:50% (vol/vol) glycerol-PBS (0.05 M Na_2_HPO_4_, 0.85% NaCl, pH 7.5) with 1% *p*-phenylenediamine, and the virus-like particles were enumerated under epifluorescence microscope (Olympus BX 41, USA). Prokaryotes were distinguished from virus-like particles (VLPs) on the basis of their relative size and brightness [18]. A blank was routinely examined as a check for contamination of the equipment and reagents.

Total viable counts (TVC) were measured to estimate the physiologically active bacteria [19]. Briefly, 5 ml water sample was mixed with 50 μl of 0.05% yeast extract, 50 μl of antibiotic cocktail (nalidixic acid, pipemidic acid, piromidic acid, and cephalexin). This antibiotic cocktail acts as a specific inhibitor of DNA synthesis and prevents cell division without affecting other cellular metabolic activities. The resulting cells continue to metabolize nutrients and become elongated. After incubation in dark for 6 hours, the samples were fixed in 2% formalin, stained with 100 μl of acridine orange (0.01%) and enumerated using an epifluorescence microscope.

### Transmission electron microscope (TEM) analysis

#### Viral lytic infection and viral infected prokaryotic cell morphotypes

Prokaryote cells contained in formalin-fixed water samples (final conc. 2% v/v) were collected on triplicate TEM grids (400-mesh, carbon-coated Formvar film) by ultracentrifugation (Optima LE-80K, Beckman Coulter SW40 Ti Swing-Out-Rotor at 70,000 × *g* for 20 min at 4°C) according to Pradeep Ram et al. [20]. Each grid was stained at room temperature (ca. 20°C) for 30 s with uranyl acetate (2%, pH = 4), rinsed twice with 0.02 μm-filtered distilled water to remove excess stain, and dried on filter paper. Grids were examined using a JEOL 1200Ex TEM operated at 80 kV and a magnification of 20,000 to 60,000 × to distinguish between prokaryote cells with and without intracellular viruses. A prokaryote was considered infected when at least five viruses, identified by shape and size, were clearly visible inside the host cell. At least 400–600 prokaryote cells were inspected per grid to determine frequency of visibly infected prokaryote cells (FVIC). FVIC counts were converted to the frequency of infected cells (FIC) using the following formula: FIC = 9.524FVIC– 3.256 [21]. The FIC was then converted to viral-induced prokaryote mortality (VIPM, as a percentage per generation) using the following equation, VIPM = (FIC+0.6×FIC2)/ (1–1.2×FIC) [22].

Viral infected prokaryotic cell morphotypes were subjectively recorded as elongated thin rod, short rod, fat rod, filamentous and cocci (i.e. prokaryotic morphopopulations) based on observations during TEM examination. The above classification can however result in some overlaps among groups. To minimize this, cocci were defined as having a length: width ratio between 1 and 2, fat rods were defined as having a length:width ratio between 2 and 5 or having a width greater than 200 nm, and thin rods were defined as having a length: width ratio greater than 5 and a width less than 200 nm [23]. Burst size (BS) was estimated from the number of viruses in those visibly infected cells that were totally filled with phages, i.e. the maximum BS (BSmax) [20, 23].

#### Phenotypic diversity of viruses

For visualization of shape and diameter of viral capsids, viruses on the prepared grids were visualized by JEOL 1200EX TEM at a magnification of 65000–100000x following ultracentrifugation and uranyl acetate staining [24]. Viruses were classified as myoviruses, podoviruses, siphoviruses and non-tailed viruses based on their morphology [25].

### Statistical analysis

Differences in physicochemical and biological parameters were tested by one way analysis of variance (ANOVA). Potential relationships among variables (abiotic and biotic) were tested by linear pair-wise correlations (Pearson correlation analysis). Principal component analysis (PCA) was performed to understand the relationship between biotic and abiotic variables using PAST software (version 3). All statistical analysis was performed by using SPSS software (version 15).

## Results

### Physicochemical environment

The Cochin estuary (CE) is largely under the influence of monsoonal rains. The entire estuary is fresh water dominated during monsoon months (MON) (June-September) with high riverine influx. The average salinity during the monsoon was 3.8 ±4.2 (**Table 1**) with the lowest and highest value recorded in the southern estuary (<2) and inlet region (8.5±4.6) respectively. The salinity gradually increased in the estuary during the post monsoon period (PM) (7.5±7.2) and reached its maximum (17.6 ± 4.2) during the pre monsoon season (PRM). The seasonal fluctuation in temperature was less than 4°C with water temperature ranging from 27.5°C (MON) to 31.6°C (PRM). Throughout the study period, the water column was well-oxygenated (4.0±1.9 mg l^-1^). Nutrients showed significant (P<0.05) spatial and seasonal variations. The concentration of nutrients such as nitrate (15.3±9.5 μM), ammonia (13.9±8.1 μM) and silicate (64.0 ±39.4 μM) were generally high in the monsoon, whereas PO_4_ (1.3±1.1 μM) was found to be low and comparable during all the seasons. Chl *a* showed large variability throughout the estuary (0.6–48.1 mg m^−3^) with an average high of 17.8 ± 12.8 mg m^−3^ in the PRM than other seasons (Table 1). Like chlorophyll *a*, pheophytin also varied widely (0.9 to 16.8 mg m^−3^) with significantly (p< 0.001) high chlorophyll: pheophytin ratio in PRM (9.9±16.4) than MON (1.1 ±1.0) (**Table 1, S2 Fig**).

**Table 1:** Mean ± Standard deviation for environmental and microbial characteristics of Cochin Estuary. WT: Water temperature, Sal: Salinity, DO: Dissolved oxygen, NO_2_: Nitrite, NO_3_: Nitrate, NH_4_: Ammonia, PO_4_: Phosphate, SiO_4_: Silicate, VA: Viral abundance, PA: Prokaryotic abundance, TVC: Total viable prokaryote, VPR: Virus to prokaryote ratio, FIC: Percentage of frequency of infected prokaryotic cells, BS: Burst size mean, Chl *a*: Chlorophyll *a* and Pheo: Pheophytin. One-way ANOVA was used to test the differences in environmental and microbial parameters with seasons at a significant response considered to be at p < 0.05. NS: not significant.

Parameters (units)	MON	PM	PRM	*p* value
**WT(°C)**	27.5±1.0	29.4±0.9	31.4±1	0.001
**Sal (psu)**	3.8±3.9	7.5±7.2	17.6±3.7	0.001
**DO (mg l**^**-1**^**)**	3.3±0.5	4.1±0.7	4.5±0.29	0.001
**pH**	7.1±0.3	6.9±0.3	7.4±0.3	0.001
**NO**_**2**_ **(μM)**	0.8±0.2	0.3±0.1	3.6±2.6	0.001
**NO**_**3**_ **(μM)**	21.6±9.1	5.6±5.2	18.9±3.5	0.001
**NH**_**4**_ **(μM)**	13.9±8.0	9.4±14.6	6.9±6.9	NS
**PO**_**4**_ **(μM)**	1.5±1.1	0.7±0.6	1.7±1.3	NS
**SiO**_**4**_ **(μM)**	113.8±18.8	50.2±18.7	29.6±9.8	0.001
**Chl a (mg/m**^**3**^**)**	4.0±2.7	15.1±9.2	17.8±12.8	0.002
**Pheo (mg/m**^**3**^**)**	5.2±2.4	5.8±3.2	7.1±5.2	NS
**VA (10**^**6**^ **VLPs/ml)**	9.5±2.2	14.3±5.7	22.1±6.6	0.001
**PA (10**^**6**^ **Cells/ml)**	1.2±0.4	1.8±0.7	2.6±1.0	0.01
**TVC (10**^**6**^ **Cells/ml)**	0.2±0.1	0.4±0.1	0.5±0.2	0.001
**VPR**	8.3±2.7	5.7±2.3	13.3±5.5	0.001
**FIC %**	7.5±5.7	9.6±2.3	11.6±5.9	NS
**BS**	20.7±7.3	24.4±9.4	24.0±7.8	NS

### Viral and prokaryotic abundances

**Fig 1:**
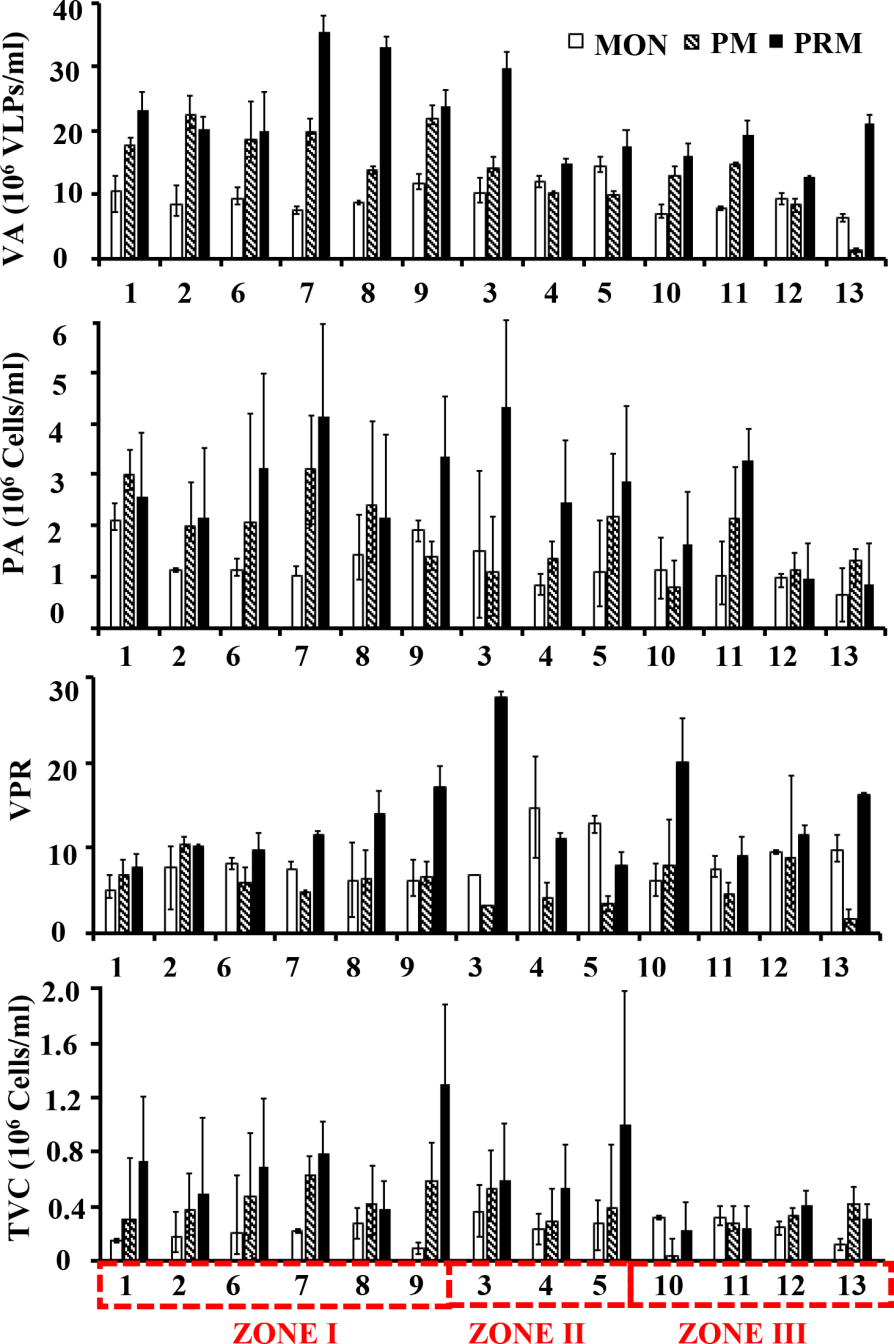
Seasonal variability in (a) Viral abundance (VA), (b) Prokaryote abundance (PA), (c) Total viable prokaryotes (TVC) and (d) Virus to prokaryote ratio (VPR). The stations are represented in the X axis and the red dotted box represents different salinity zones.

VA was highly variable (1.4–35.3 x 10^6^ VLPs mL^–1^) compared to PA (0.7–4.6 × 10^6^ cells mL^–1^) with both their minima and maxima occurring in the freshwater and euryhaline/mesohaline regions of the estuary respectively **(Fig 1)**. Overall viruses (mean = 15.3x 10^6^ VLPs mL^–1^) predominated bacteria (mean = 1.89 x 10^6^ cells mL^–1^) by an order of magnitude. Pronounced seasonal patterns were observed in both VA and PA with their highest abundances in the PRM (**Table 1**). Large variability in the abundance of viruses (Coefficient of Variation CV% = 47.9%) and prokaryotes (CV% = 49.7%) was reflected in the virus to prokaryote ratio (VPR) which ranged from 2.8 to 27.70. The distribution of viruses and prokaryotes was more or less similar among the lakes resulting in a strong correlation (r = 0.43, p < 0.01) between the two variables, indicating that prokaryotes were the dominant hosts for viruses. Total viable counts ranged between 0.1–1.0 × 10^6^ cells mL^–1^ which represented 15.1% to 23.8% of the total prokaryotic counts with minimum and maximum abundances observed in the PRM and MON season respectively. VA showed significant positive correlation with chlorophyll *a* (r = 0.34, p<0.05) and total viable counts (r = 0.56, p<0.01).

### Viral infection and burst size estimates

**Fig 2:**
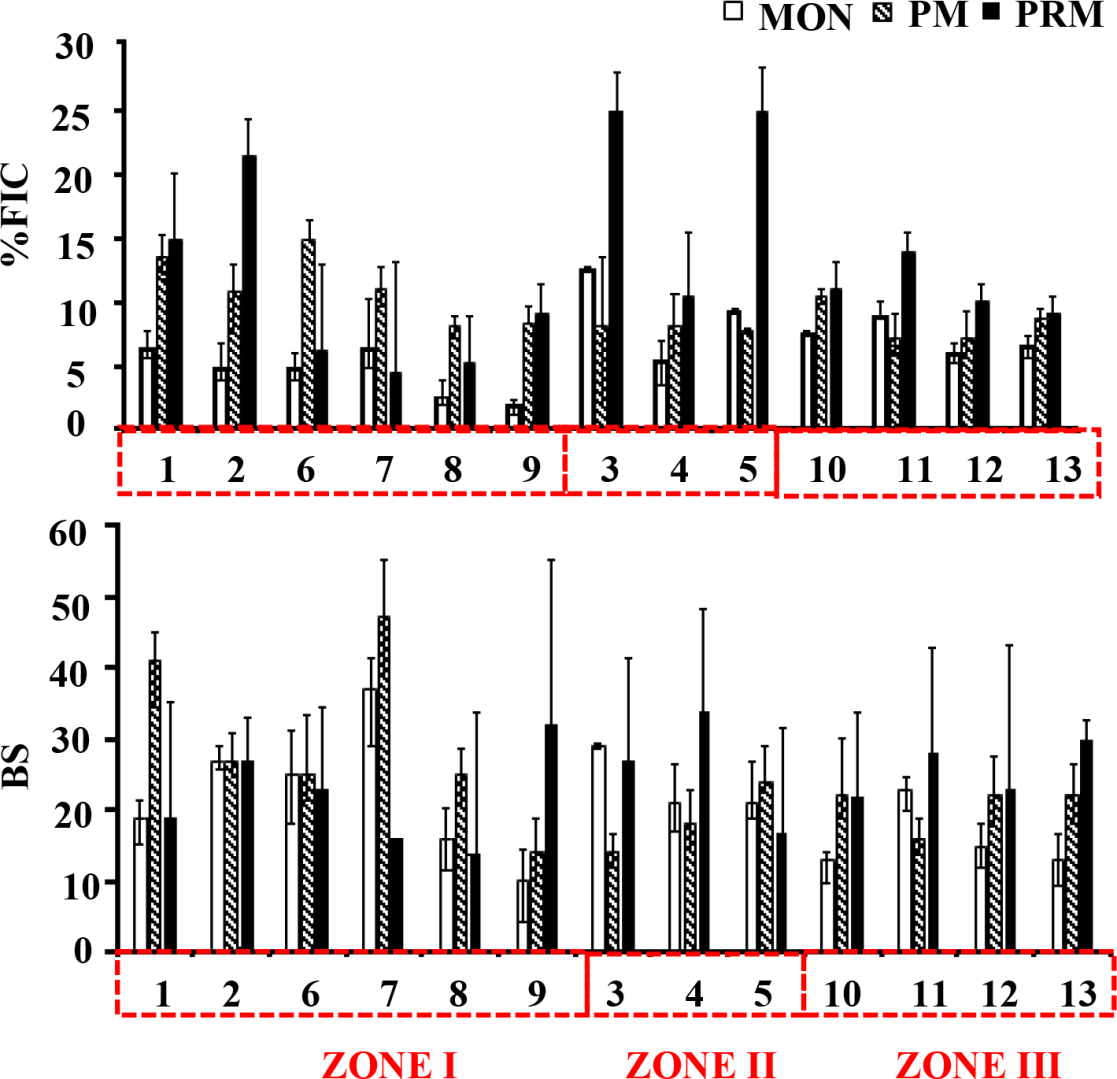
Seasonal variability in (a) the percentage of viral infected prokaryote cells (FIC) and (b) Burst size (BS) estimates. The stations are represented in the X axis and the red dotted box represents different salinity zones.

Frequency of visibly infected cells (FVIC) estimates by transmission electron microscopy provide direct evidence of phage infection and facilitates the calculation of virus-induced prokaryote mortality (VIPM) rates. The frequency of virus-infected cells (FIC) calculated from FVIC varied with time and space in Cochin Estuary. FIC varied by a factor of 13 and ranged between 1.9% and 25.3% (**Fig 2**). The high infection rate was observed in the PRM at euryhaline region of the estuary (Station 3) coinciding with viral and prokaryote maxima and corresponded to 42% of viral induced prokaryote mortality. FIC was correlated significantly (p < 0.05) with VA and BA, but none of the measured abiotic parameters (**Table 2**). The burst size (BS) of individual cells varied over two orders of magnitude, ranging from 6–135 with minimum during MON (mean = 21±7) **(Fig 2)**. The BS increased with increase with salinity in the estuary during PM (mean = 24±9 viruses prokaryote^-1^) and PRM (mean = 24±7 viruses prokaryote^-1^). Like FIC, one way ANOVA indicated BS to vary seasonally and spatially.

**Table 2:** Pearson’s correlation coefficient (r) between different variables in Cochin Estuary (n = 49).

	VA	PA	TVC	FIC	BS	WT	pH	SAL	DO	NO_2_	NO_3_
**PA**	0.43**										
**TVC**	0.55**	0.34*									
**VPR**	0.5***	-0.42**	-0.30*								
**FIC**	0.37**	0.36*									
**BS**		-0.27*	-0.29**	0.33*							
**WT**		0.42**									
**pH**	0.45**		0.40**								
**SAL**	0.50***	0.30*	0.47**				0.6***				
**DO**		0.31*				0.70***					
**NO**_**2**_	0.38*										
**NO**_**3**_		-0.34*				-0.60***			-0.50***	0.31*	
**NH**_**4**_					0.38**					-0.30*	
**PO**_**4**_							0.50***		-0.40**		0.32*
**SiO**_**4**_	-0.60***	-0.41**	-0.47**			-0.60***	-0.31*	-0.60***	-0.60***	-0.36*	0.38*
**Chl a**	0.34*	0.32*	0.31**			0.38*	0.30*	0.49**	0.32*		-0.45**

VA: Viral abundance, PA: Prokaryotic abundance, TVC: Total viable prokaryotes, VPR: Virus to prokaryote ratio, FIC: Percentage of frequency of infected prokaryotic cell, BS: Burst size mean, WT: Water temperature, DO: Dissolved oxygen, NO_2_: Nitrite, NO_3_: Nitrate, NH_4_: Ammonia, PO_4_: Phosphate, SiO_4_: Silicate and Chl *a*: Chlorophyll *a*.

Levels of significance:

* p<0.05

** p<0.01 and

*** p<0.001.

### Viral infection of different prokaryotic cell morphotypes

The viral morphotypes which were determined based on observation during transmission electron microscopy examination mainly belonged to *Myoviridae* (non-enveloped viruses with contractile tail), *Siphoviridae* (long non-contractile tail), *Podoviridae* (short non-contractile tails) and non-tailed viruses. Among them, Myoviruses were the most dominant (44%) followed by non-tailed (24%), siphoviruses (17%) and podoviruses (15%) which did not exhibit much seasonal variations in the CE. The diameter of the intracellular viral capsid ranged from 20 to 100nm with an average of 40 ± 12nm (n = 240). Among the size range, >85% of the head size diameter of ‘extra cellular phage particles’ and intracellular phages was ≤ 60 nm, suggesting that they were typical bacteriophages.

**Fig 3:**
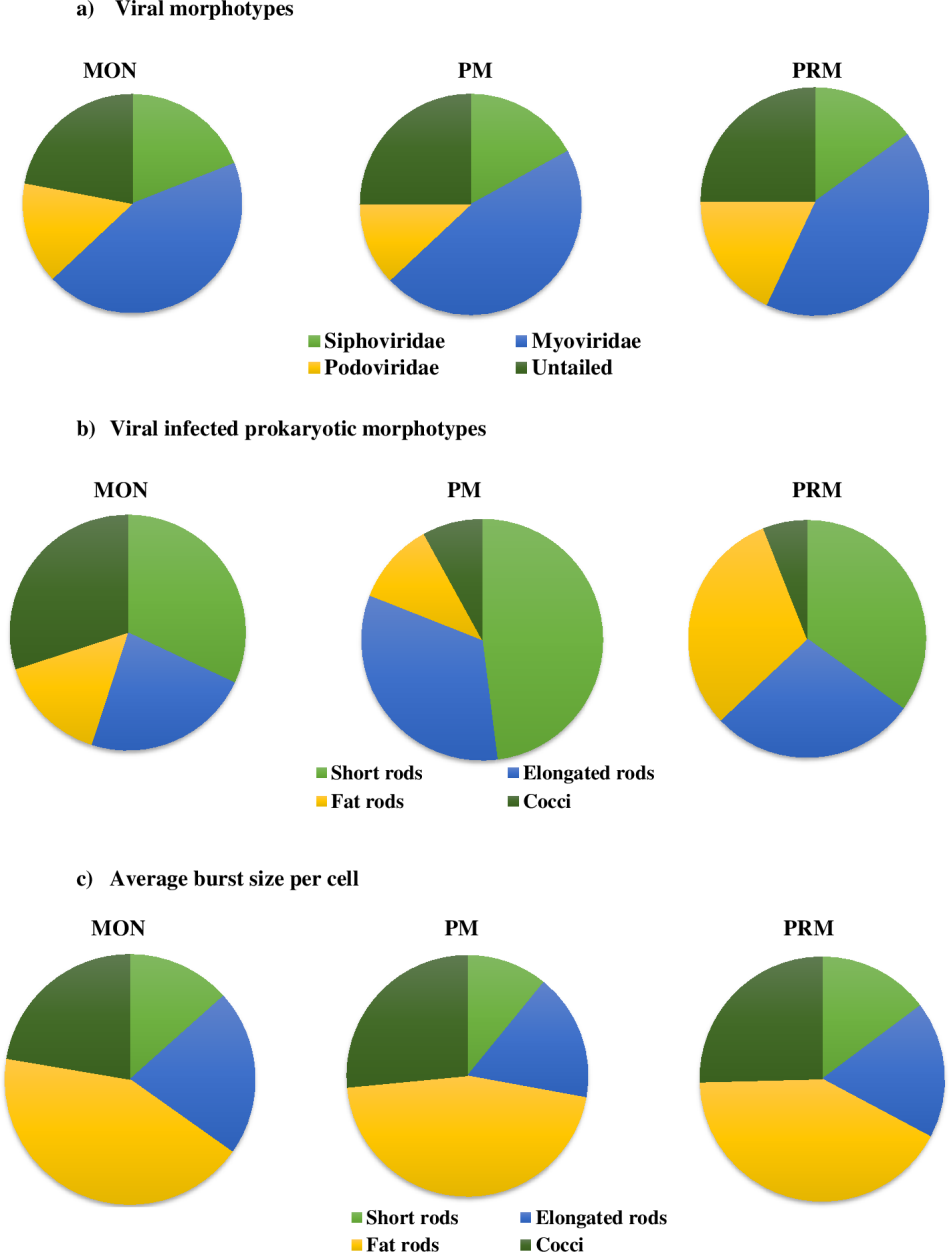
Seasonal variations in (a) Viral morphotypes, (b) Viral infected prokaryotic morphotypes and (c) Average burst size estimates.

Our observation and evaluation of 4 observed bacterial morphotypes (short rods, elongated rods, fat rods and cocci) could have led to some overlapping among the groups. Among the bacterial morphopopulations, rods were the most dominant forms representing about 80% of the entire bacterial community throughout the study period. Seasonal variability in viral infected bacterial morphotypes especially cocci was observed between monsoon (30%) and dry months (7%) **(Fig 3).** Among the rods, the fat rods were highly infected in the PRM season which coincides with increased chlorophyll *a* and salinity concentrations. The most preferred prokaryotic morphotype by viruses were short rods (48%), whereas, the least preferred morphotype was cocci (8%) during PM **(Fig 3)**. Similarly, during PRM, short rods (35%) were dominantly infected followed by fat rods (31%) and elongated rods (28%) respectively. However, during MON, fat rods were the most preferred hosts (32%), followed by cocci (30%), elongated rods (23%) and short rods (15%). However, no filamentous forms were observed during all the three seasons. The bacterial morphotypes investigated exhibited strong differences in terms of the frequency of cells containing mature phages and the BS max. Among rods, a considerable difference in BS max was observed (**Fig 3**). The fat rods recorded the highest BS (55 viruses bacterium^-1^) when compared to other morphotypes.

## Discussion

**Fig 4:**
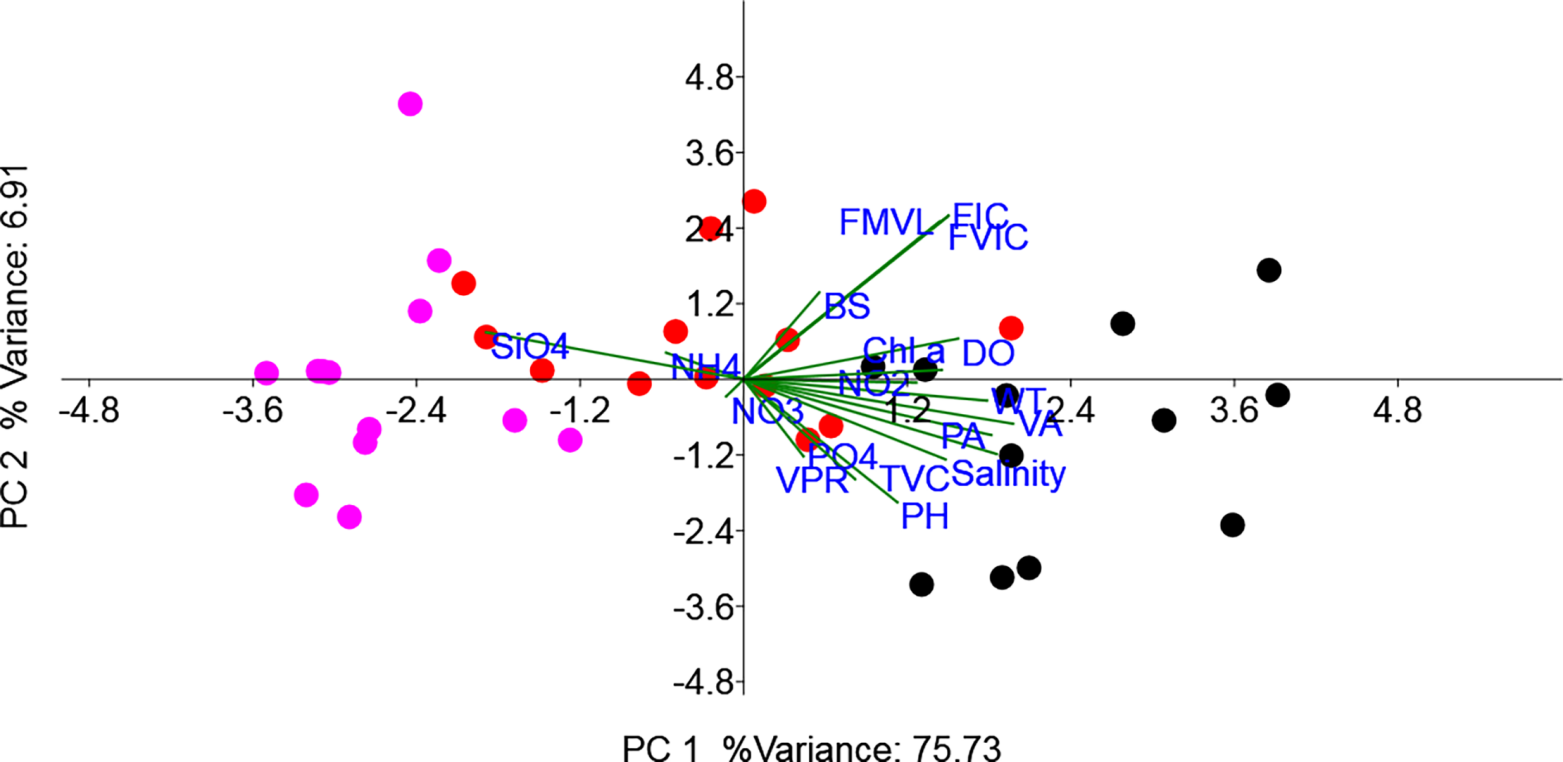
Principal component analysis (PCA) biplot representing the distribution of microbial parameters and their interrelationship of physicochemical variables. Temp: Temperature, DO: Dissolved oxygen, NO_2_: Nitrite, NO_3_: Nitrate, PO_4_: Phosphate, SiO_4_: Silicate, PA: Prokaryotic abundance, TVC: Total viable prokaryotic count, VA: Viral abundance, VPR: Virus to prokaryote ratio, FVIC: Frequency of visibly infected cells, FIC: Frequency of infected prokaryotic cells, VIPM: Viral mediated prokaryotic mortality, BS: Burst size estimates.

The present study is one among the few conducted in a monsoon impacted tropical estuarine system (Cochin Estuary) which documents the standing stock of viruses and their lytic infection in relation to environmental parameters across three distinct seasons. The abundance and distribution pattern of viruses are comparable with previous reports from Cochin Estuary [12, 13] and other estuarine systems [10, 26, 27]. Spatio-temporal patterns were evident in viral abundance and in their infection rates of prokaryote morphopopulations between the wet (monsoon) and dry (pre- and postmonsoon) seasons which were related to primary and secondary production. Such seasonal fluctuations in the viral abundance and their infection rates are more prominent in freshwater and estuarine systems due to the shift in the host composition and diversity which are principally regulated by prevailing environmental parameters [28]. In the present study high standing stock of viruses and bacteria accompanied lytic infection rates in the euryhaline/mesohaline region of the estuary suggests salinity to have an influential role in driving interactions virus-prokaryote interactions (PCA biplot, **Fig 4**), similar to those observed in other estuarine systems [9, 29]. Such a potential importance of salinity on viral-host interaction is particularly meaningful for marine systems, where the requirement of specific ions (such as sodium and potassium) and certain divalent cations for bacteriophages (to remain infective) and the prokaryote hosts (to enhance phage adsorption on their cell surface) respectively, are requisites for successful viral infection and proliferation [30]. Moreover in such environments, there exists a possibility that salinity could favor the transition of lysogenic to lytic resulting in the production of viral particles in the bacterial host through high rate of spontaneous prophage induction as previously evidenced in ɸHSIC prophage infecting *Listonella pelagia* [31].

**Fig 5:**
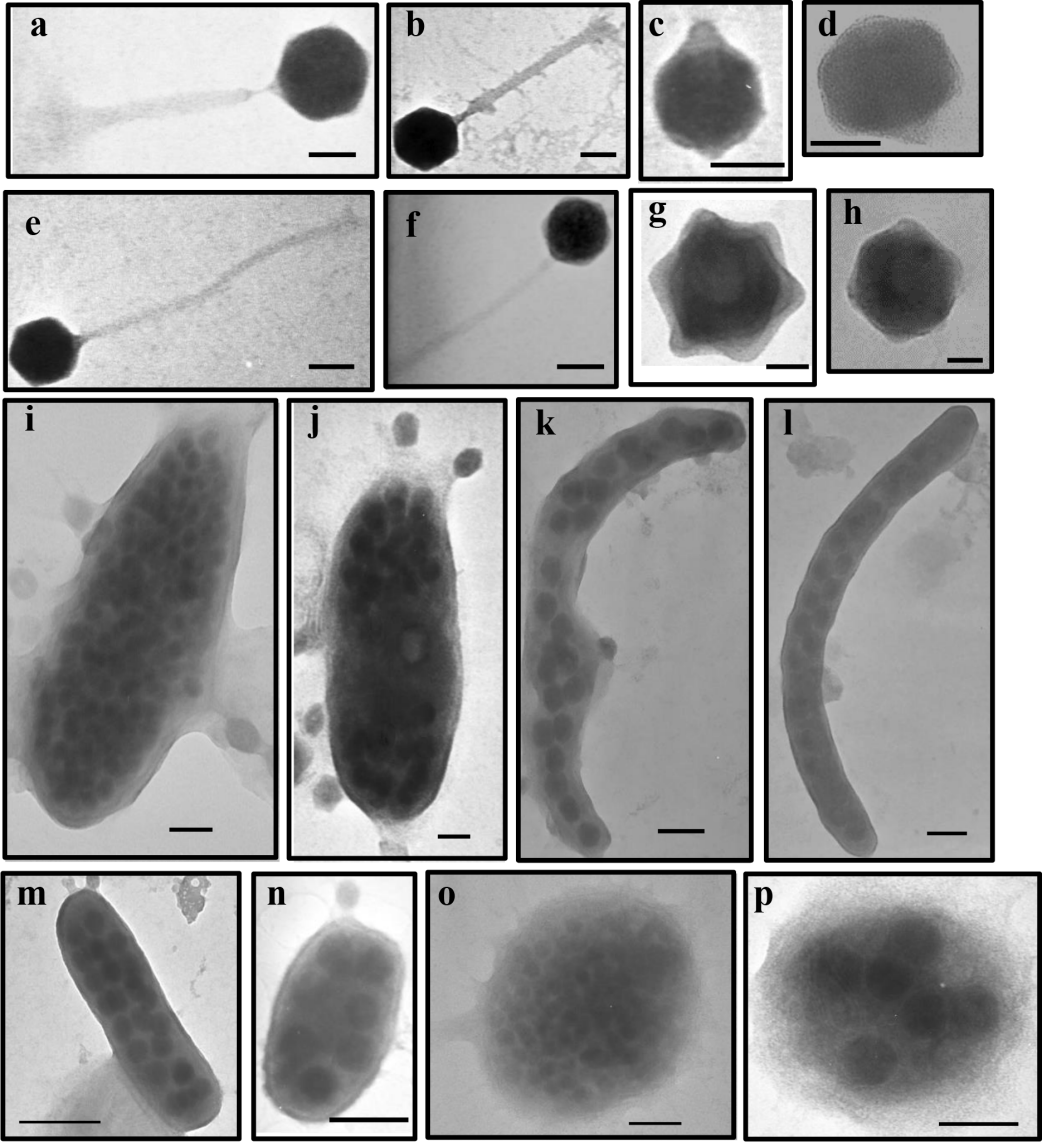
Transmission electron micrographs of viral morphotypes belonging to *Myoviridae* (a, b), *Podoviridae* (c, d), *Siphoviridae* (e, f) and non-tailed viruses (g, h); scale bar = 50 nm, and viral infected prokaryotes belonging to different morphotypes such as fat rods (i, j), elongated rods (k, l), short rods (m, n) and cocci (o, p), scale bar = 100nm.

Analysis of viral morphology by TEM in Cochin estuary yielded tailed viruses which belonged to the three families of the order Caudovirales (Myoviridae, Siphoviridae and Podoviridae) in addition to non-tailed viruses (**Fig 5A**–**5H**). Previous studies conducted in marine systems have linked phage morphotypes to varying environmental gradients with salinity identified to be an important variable [32]. In this tropical system, the dominance of phages with capsid size of ≤60nm suggest that they were typical bacteriophages, as previously observed from several marine [33] and freshwater systems [2]. Because ‘lifestyles’ among tailed phages differ, morphology provides clues about host range and viral replication. Irrespective of the seasons, myoviruses (contractile tails) was the dominant viral morphotype, representing 43% of the total population in Cochin Estuary. This group belonging to the double stranded DNA bacteriophage family was associated with relatively high lytic infection, which is in agreement with earlier reports from other estuarine systems [34, 35]. Myoviruses are known to have a broader host range than other phages, even infecting different species of bacteria [25, 36], and to benefit from increased generation times in host populations [33]. In contrast to myoviruses, the low percentage of podoviruses (short non-contractile tail) and siphoviruses (long flexible tails) in our study are often described to have narrow and intermediate host range respectively [33]. By contrast, many siphoviruses can archive their genomes in host cells, tying their replication rate to that of the host, until an environmental cue triggers the lytic cycle [33].

In our study a significant percentage of non-tailed phages comprising of 22–25% of viral assemblages was observed. Although large capsid diameter of ≥150 nm belonging to family Phycodnaviridae are known to infect eukaryote algae, the high percentage of non-tailed viruses with capsid diameter of ≤60nm is intriguing. Other factors such as tail loss during centrifugation and deposition of podoviruses on their tails when deposited onto the grids cannot be rule out; henceforth the percentage of siphon- and podoviruses reported in our study could perhaps be underestimates. It has been reported that marine viruses may lose their tail through natural decay, which in case could result in similar capsid diameter between tailed and non-tailed viruses. Further studies on the above lines would substantiate the role and importance of non-tailed viruses in such tropical systems.

We used TEM (i.e whole cell examination) method for the determination of viral infection and burst size estimates. TEM method not only facilitates the calculation of viral induced prokaryotic mortality but also allows a relatively easy comparison of viral infections among aquatic systems. Discussions about interpreting the estimates of bacterial mortality due to viral lysis based on whole cell approach are detailed in [37], and thus, will not be reiterated herein. In the present study, burst size was positively correlated with the viral infection and negatively with prokaryote abundance, suggesting that high viral release should have occurred under high prokaryotic production. Incidence of high viral infection rates through the possibility of spontaneous prophage induction resulted up to 42% of prokaryotic mortality during pre-monsoon season suggesting that the lytic mode of infection is important in Cochin estuary. This is further substantiated by a high virus to prokaryote ratio suggesting that maintenance of a high number of viruses is dependent on the most active fraction of bacterioplankton [20]. In most of the study stations especially during the dry seasons, the viral infection rates were above the threshold level of 10% which has been reported to impact prokaryote community structure [38] and their carbon metabolism [7] in freshwater systems. Therefore variable viral induced prokaryote mortality rates that were observed in the dry and wet seasons can perhaps influence the carbon flow to higher trophic level. Lower viral mediated prokaryote mortality in the monsoon compared to non-monsoon months did not result in considerable percent lysogeny (undetected to 0.8%, Jasna, personal communication) by mitomycin C induction, thus contrasting with other studies from tropical systems where the estuarine gradient from fresh water to sea water led to the emergence of lysogenic prokaryotes [39, 40].

Studies on the potential effects of viral lysis on different prokaryotic morphotypes (i.e. the so-called prokaryotic morphopopulations) and cell size distribution are limited, although these variables directly determine prokaryotic biomass in aquatic systems. Proakaryotes can modify their morphology in response to environmental cues, and selective forces such as nutrient uptake capabilities and predation have been shown to have a strong impact on bacterioplankton [15]. The strong differences in the frequencies of prokaryotic cells filled with mature phages (i.e., BS_max_) and of viral infection among the investigated prokaryotic morphopopulations (**Fig 5I**–**5P**) might be due, at least to some extent, to the physiological differences associated with different prokaryotic phenotypes. The average burst size reported in our study (mean = 21 viruses prokaryote^-1^) is lower than the reported values for marine systems [41], which was due to the dominance of viral infected short rod cells which are known to have relatively lower burst size estimates owing to their lower cell volume. Studies have indicated bacterial cell volume to play a decisive role in supporting higher burst sizes [23, 20]. In the present study, the abundances of dominant and most infected rod shaped prokaryote were well above the threshold level of 2 x 10^5^ cells ml^-1^, necessary for the occurrence of detectable phage infection in the plankton [42]. We consider that selective lysis of cells belonging to a particular morphotype, may induce substantial changes in the functional roles of natural prokaryotic community and biogeochemical processes.

Overall in Cochin estuary, viruses had differential impact on prokaryotic morphopopulation with seasons (wet versus dry), which can have variable influence on the carbon and energy flows in this tropical ecosystem. The constant supply of host cells (10^6^ ml^-1^) and relatively high phage particle abundance (10^7^ per ml) in this estuarine system could fit in the criterion of pseudolysogeny, where certain lysogens can develop pseudolysogenic interactions due to unstable repressor protein, eventually responsible for the high rate of spontaneous induction [31]. Among the factors, salinity was a significant factor in driving virus-prokaryote interactions. Preferential viral infection could be related to the physiological state and metabolic activity of the host prokaryote cells, where successful lysis can have direct impact on bacterial community structure [38], however the above hypothesis needs to be tested in future investigations to better understand the functional significance of viruses in Cochin estuary.

## Supporting information

S1 Fig Sampling locations in Cochin estuary. Stations 1 and 6 are the two inlets. Stations 1, 2, 7, 8 and 9 represent the central estuary, stations 3, 4 and 5 represent the north estuary and stations 10–13 represent the south estuary. The boxes represent the different salinity zones (I, II and III) in the estuary.
(DOCX)

S2 Fig The zonal average of biological parameters in MON, PM and PRM. (a)VA-Viral abundance,(b) PA- Prokaryotic abundance,(c) TVC-Total viable prokaryotic count, (d)VPR- Virus to prokaryotes ratio, (e) FIC—Percentage of infected prokaryotic cell, (f) BS-Burst size mean.
(DOCX)
