# Supplementary figures and images for: Differential impact of lytic viruses on prokaryotic morphopopulations in a tropical estuarine system (Cochin estuary, India)
Source: PLoS One. 2018 Mar 13;13(3):e0194020. doi: 10.1371/journal.pone.0194020 (PMC5849291; doi:10.1371/journal.pone.0194020)

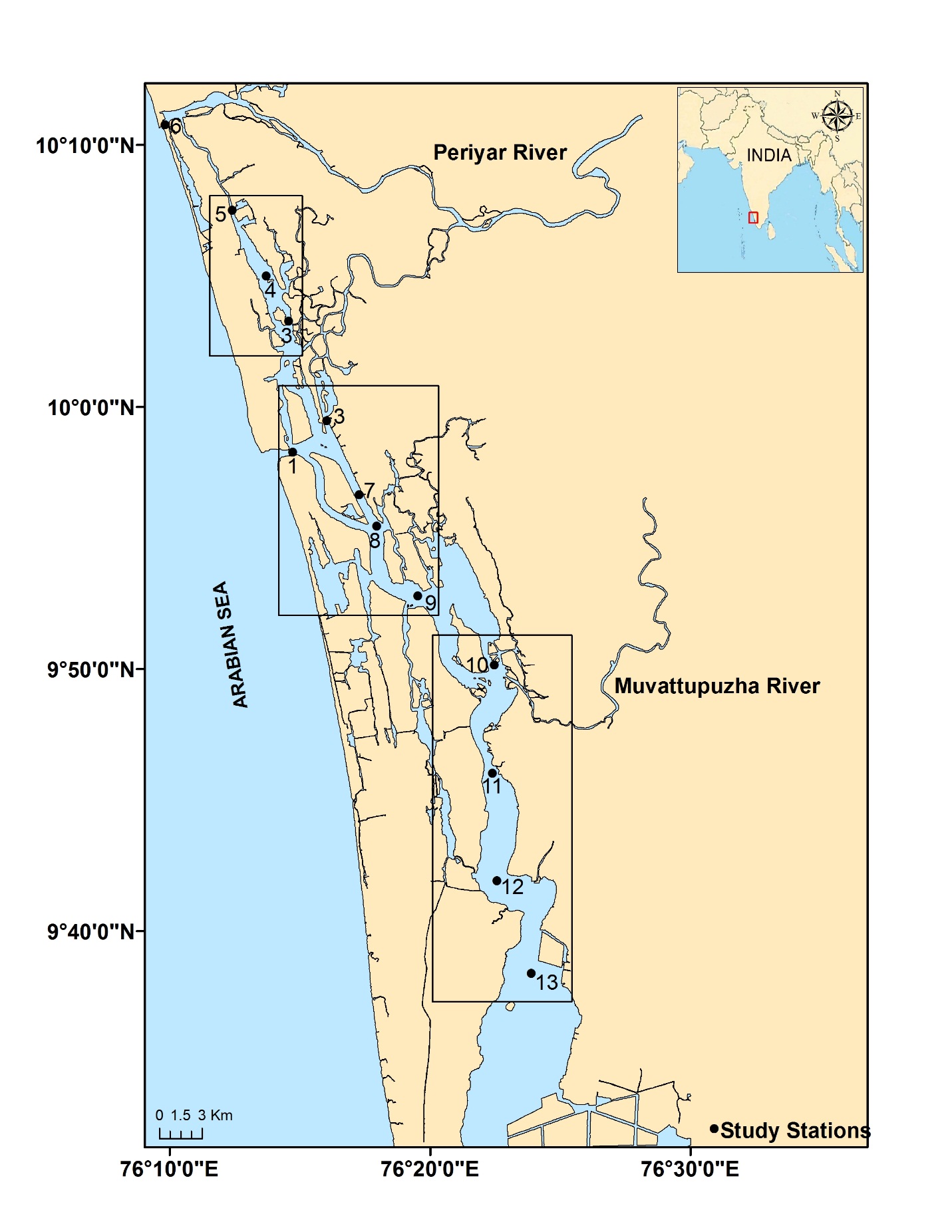


Zone III

Zone II

Zone I

Supplement: S1 Fig — Stations 1 and 6 are the two inlets. Stations 1, 2, 7, 8 and 9 represent the central estuary, stations 3, 4 and 5 represent the north estuary and stations 10–13 represent the south estuary. The boxes represent the different salinity zones (I, II and III) in the estuary. (DOCX) [file pone.0194020.s001.docx]
