# Supplementary material for: Differential impact of lytic viruses on prokaryotic morphopopulations in a tropical estuarine system (Cochin estuary, India)
Source: PLoS One. 2018 Mar 13;13(3):e0194020. doi: 10.1371/journal.pone.0194020 (PMC5849291; doi:10.1371/journal.pone.0194020)
Supplement: S2 Fig — (a)VA-Viral abundance,(b) PA- Prokaryotic abundance,(c) TVC-Total viable prokaryotic count, (d)VPR- Virus to prokaryotes ratio, (e) FIC—Percentage of infected prokaryotic cell, (f) BS-Burst size mean. (DOCX) [file pone.0194020.s002.docx]

**
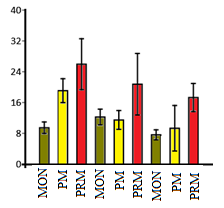

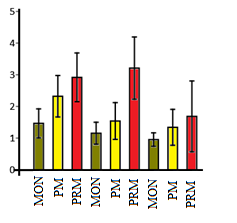

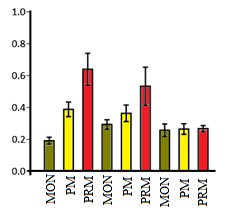
**

**% FIC**  **PA (10^6^Cells/ml)**

**BS TVC (10^6^Cells/ml)**

1. **(b) (c)**

**VPR VA (10^6^VLPs/ml)**

**(d) (e) (f)**


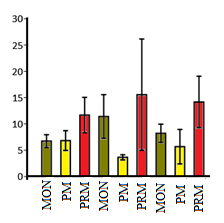

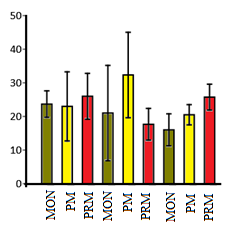

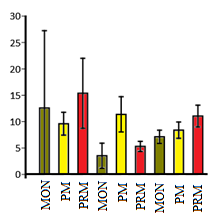


**Zone I Zone II Zone III Zone I Zone II Zone III Zone I Zone II Zone III**
